# Health Benefits of Fermented Bamboo Shoots: The Twenty-First Century Green Gold of Northeast India

**DOI:** 10.1007/s12010-021-03506-y

**Published:** 2021-01-26

**Authors:** Prapti Behera, Seetharaman Balaji

**Affiliations:** Department of Biotechnology, Manipal Institute of Technology, Manipal Academy of Higher Education, Manipal 576104 Karnataka, India

**Keywords:** Bamboo shoot, Northeast India, Fermentation, Microorganisms, Health benefits

## Abstract

The word “bamboo” reminds us of “a hollow stick,” but it is filled with a plethora of health benefits. The tribals of northeastern India ferment these beneficial bamboo shoots for the goodness of mankind. Fermentation is an important age-old biotechnological procedure used for the preservation of food products. Fermented bamboo shoots form the niche for many microorganisms, and this confers positive effects and advantages in many ways. These magical shoots have tremendous health benefits like anti-cancer, anti-oxidant, anti-aging, cardioprotective, weight loss, probiotics, to name a few. Apart from health benefits, fermented bamboo shoots form important functional foods and have industrial and economical values. Though these are commonly found and started in the tribal area, and local markets, today, they are valuable all around the world, as popular as gold. Hence, fermented bamboo shoots are referred as “green gold” of India. This review briefs about various health benefits, advantages, disadvantages, future scope, and finally the economic values of fermented bamboo shoots, the “green gold” of the twenty-first century.

## Introduction

Fermentation may be defined as a method of generation of a product, by culture of mass of microorganisms [1]. People have been using fermented foods for a very long time. Fermented foods form a very important part in our lives and use biotechnology as a tool to produce and preserve for a long time. During the production of fermented foods, enzymes or microbes are used, which lead to required biochemical changes and important changes in the food. These fermented foods have been produced and consumed approximately 5000 years back, concurrently alcohol fermentation from barley, and wine from grape were produced. Microorganisms such as bacteria, molds, and yeasts were used. For instance, lactic acid bacteria, *Aspergillus* spp., and *Saccharomyces* spp., respectively. Globally, fermented foods and beverages ranges from 20 to 40% of the food supply. The fermented foods such as bread, wine, cheese, yogurt, idli, and dosa [2] are common in many countries such as Europe, India, North Africa, and the Middle East.

The table shows the fermented foods from different parts of the world (Table 1).

**Table 1:** Fermented foods worldwide (adapted from [2])

Group	Food examples	Regions of production
Dairy	Cheese; Yogurt: kefir, kurut	Europe, North America, Middle East
Beverages	Beer, coffee, tea, cocoa, wines, cider	All parts of the world
Cereals	Rolls, bread, doughnuts, idli, dosa, pancakes, tape ketan	All parts of the world
Fish	Bagoong, paak, fish sauce	Eastern Asia, South-East Asia, Europe
Meat	Salami, jerky, pepperoni, pickled meat, country ham	Middle East, Europe, India, North America
Legumes	Soy sauce, miso	India, South-East Asia

Bamboo shoots are fermented in northeast India by the tribal people, due to their plethora of health benefits with high medicinal value and immense food value. They are a storehouse of many important microorganisms especially the lactic acid bacteria (LAB) and yeast strains. They give the color, aroma, flavor, taste, and texture to the food product. They can be used as functional foods as they are rich in probiotics. Many fermented bamboo shoot-based food products are produced by the local tribes of northeastern India, of which few are readily available in the local market, like soidon, soibum, mesu, ekung, lung-siej, hirring, and eup*.* These fermented bamboo shoot food products, along with their nutritional value, mode of preparation, and the presence of microorganisms have been described here. This review is to account for the benefits of fermented bamboo shoot-based foods of northeast India. 

As this review lists the various advantages of fermented bamboo shoots, especially the health benefits, it is very much required to prove why fermented bamboo shoots are important and thus, how they increase the economic value and that is why it is called the “green gold” in India in this twenty-first century. Bamboo being green in color and is as valuable as gold due to the plethora of benefits, it bestows on mankind.

## Fermented Foods of Northeast India

Northeast India consists of eight states, namely, Arunachal Pradesh, Assam, Manipur, Meghalaya, Mizoram, Nagaland, Sikkim, and Tripura. It consists of a variety of people having diverse ethnic backgrounds. Most people are tribal about 75% of the population. These tribes have great knowledge about plants, forests, and edible food products from plants. They use fermentation techniques for improving food processing, preservation, and taste enhancement. In the fermented foods, microbes such as *Lactobacillus* sp., *Bacillus* sp., *Candida* sp., and *Saccharomyces cerevisiae* are present especially in *Kinema*, *Hawaijar*, *Tungrumbai*, *Bekang*, *Peruyyan*, *Soibum*, *Soidon*, *Mesu*, *Soijim*, *Ekung*, *Hirring*, *Ngari*, *Hentak*, *Tungtap*, *Gnuchi*, *Gundruk*, *Sinki*, *Ziang-sang*, *Goyang*, *Khalpi*, *Ipoh*, *Atingba*, *Kodo ko Jaanr*, and *Zutho* [1].

### Fermented Bamboo Shoots

Fermentation is used to preserve food products in Manipur [3]. There are many fermented bamboo shoot-based food products such as mesu, soibum, soidon, soijim, ekung, heccha, eup, hirring, lung-seij, tuaithur, soidonmahi, tabah bam shoot pickle, naw-maidong, jiang-sun, and soibum [4].

Bamboo shoots are edible. Fermented bamboo shoots are important to human life due to their high nutritional and medicinal value. The bamboo shoots also form an integral part of the tribal diet. They are high in dietary fiber and mineral content, low in fat, and are economically important. Traditional medicine systems like Ayurveda uses bamboo shoots as medicines for many diseases [4].

They are rich in xylan or xylooligosaccharides. Some of the medicinal applications and health benefits are as follows: anti-oxidant, anti-cancer, anti-aging, anti-free radical, weight loss, prevents cardiovascular diseases, improves digestion, anti-microbial activity due to the presence of different glycosides, flavones, and also decreases blood pressure [4].

### Microflora of Fermented Bamboo Shoots

Fermented bamboo shoot products like soidon, soibum, soijim, lung-siej, and bamboo shoot pickle samples undergo natural fermentation by *Lactobacillus* sp*.* [5].

These foods act as a storehouse of a group of gram-positive bacteria namely, lactobacillus (LAB) species [4]*.* These bacteria add flavor, aroma, and sour taste to the fermented foods. *Soidon* mainly has lactobacillus sp. such as *L. curvatus* and *Lactococcus lactis.*

Lung-siej mainly has *Leuconostoc fallax*, *L. mesenteroides*, *Lactobacillus brevis*, *L. curvatus*, and *Lactococcus lactis* [6].

The list of fermented bamboo shoot-based products and the associated microorganisms is shown in Table 2.

**Table 2:** Microflora in fermented bamboo shoots

Fermented bamboo shoot products	Microorganisms present	Reference
Soibum	*Lactobacillus brevis*, *L. plantarum*, *Leuconostoc fallax*, *L. mesenteroides*	[7–9]
Mesu	*Enterococcus faecium*, *Lactobacillus plantarum*, *Lactococcus lactis*	[8, 10]
Soidon	*Lactobacillus brevis*, *L. curvatus*, *L. Plantarum*, *Leuconostoc fallax, Lactococcus lactis*	[8–12]
Soijim	*Lactobacillus brevis*, *Leuconostoc fallax*, *L.mesenteroides*, *L. lactis*	[9]
Lung-seij	*Lactobacillus brevis*, *L. curvatus Leuconostoc mesenteroides*, *L. fallax*, *Lactococcus lactis*,	[9]
Ekung	*Lactobacillus plantarum*, *L. brevis*, *L. casei*, *Tetragenococcus halophilus*	[13]
Eup	*Lactobacillus plantarum*, *L. fermentum*	[13]
Hirring	*Lactobacillus plantarum*, *Lactococcus lactis*	[13]

Some examples of northeast Indian fermented bamboo shoots have been discussed below:

### Soibum

Manipur is a center for different types of fermented food products, especially prepared by *Meiteis*, the inhabitants of Manipur [18]. Soibum is a traditional fermented bamboo shoot specific to Manipur. The soibum is whitish, tastes sour, and it is prepared from the tender bamboo shoots such as *Bambusa tulda* (*Utang*), *B.balcona* (Ching saniebi), *Wanap, Unap, Dendrocalamus hamiltonii*, *Pecha*, *Melacona bambusoide* (Moubi/Muli), *Bambusa tulda* (*Utang*), and *B.balcona* (Ching saniebi), by natural fermentation. The outer casings of young shoots are removed, and the inner part is cut into pieces and washed and fermented for 3–12 months. The Meitei women make this in Manipur. It is eaten with steam rice as a normal dish by the Meitei tribe. Soibum is commonly sold as a vegetable in the market by the Meitei women in Manipur. It is rich in microorganisms such as *Lactobacillus plantatum*, *L.brevis*, *L. coryniformis*, *L. delbrueckii Leuconostic fallax*, *L. mesenteroides Lactococcus lactis*, *Streptococcus lactis*, *Enterococcus durans*, *Bacillus subtilis*, and yeasts like *Candida*, and *Saccharomyces,* to name a few [11, 13].

The nutritional value of soibum includes, moisture: 92%, acidity: 0.98%, pH: 3.9, fat: 3.2% DM, carbohydrate: 47.2% DM, protein: 36.3% DM, food value: 362.8 kcal/100 g DM, Ca: 16.0 mg/100 g, K: 212.1 mg/100 g, and Na: 2.9 mg/100 g [13].

**Fig. 1:**
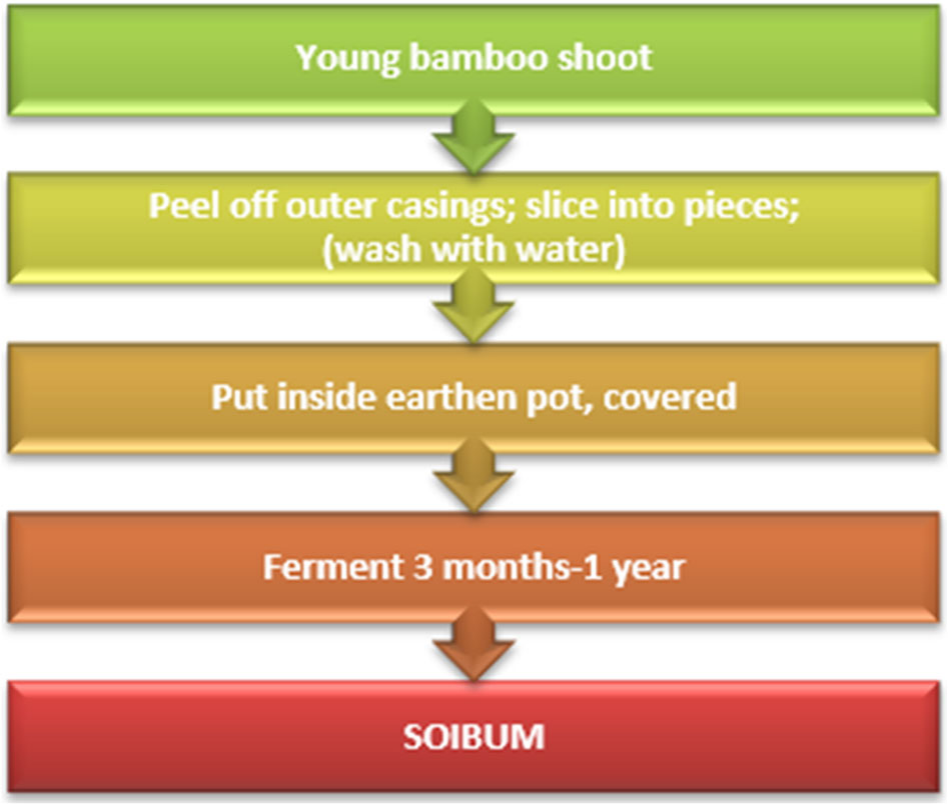
Preparation of soibum in Manipur (adapted from [11])

The preparation of soibum has been represented as a flow chart in Fig. 1.

As shown in Fig. 1., the mechanism of action of some microorganisms like *Lactobacillus plantarum*, confers antimicrobial property and increases the shelf- life of soibum [19].

### Mesu

Mesu is a common fermented bamboo shoot that is made and eaten by Gorkha tribe of Sikkim. To prepare this, edible bamboo shoots *karati bans* (*Bambusa tulda*), *choya bans* (*Dendrocalamus hamiltonii*), *and bhalu bans* (*Dendrocalamus sikkimensis*) are used. The shoots are defoliated and chopped and pressed tightly, into a hollow bamboo stem. The tip of the vessel is covered tightly with leaves and allowed to ferment naturally under anaerobic conditions for 7–15 days. Mesu is consumed as a pickle. Usually, microorganisms found in *Mesu* are *Lactobacillus brevis*, *L. plantarum*, *L. curvatus Leuconostoc citreum*, and *Pediococcus pentosaceus* [13]. Mesu has a typical flavor and taste. Mainly it is produced by the *Limboo* women of Nepal. Mesu in green bamboo vessel is sold in rainy season in the local markets of Sikkim and Darjeeling hills by the *limbo* women [11].

The nutritional value of mesu includes moisture: 89.9%, acidity: 0.88%, pH: 3.9, ash: 15.0% DM, protein: 27.0% DM, fat:2.6% DM, carbohydrate: 55.6% DM, Ca: 7.9 mg/100 g, K: 282.6 mg/100 g, Na: 2.8 mg/100 g, food value: 352.4 kcal/100 g DM [13].

**Fig. 2:**
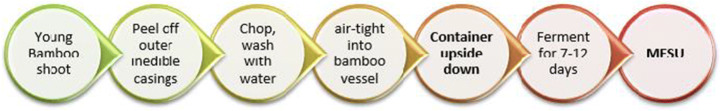
Preparation of mesu in hills of Darjeeling (adapted from [11])

The method of preparation of mesu has been illustrated in Fig. 2.

Mechanism of action of different microorganisms occurs at the stage where the chopped bamboo shoots are kept in air-tight bamboo vessels and for fermentation for 7–12 days, gives the texture and color to the mesu food product (refer Fig. 2) [19].

### Soidon

**Fig. 3:**
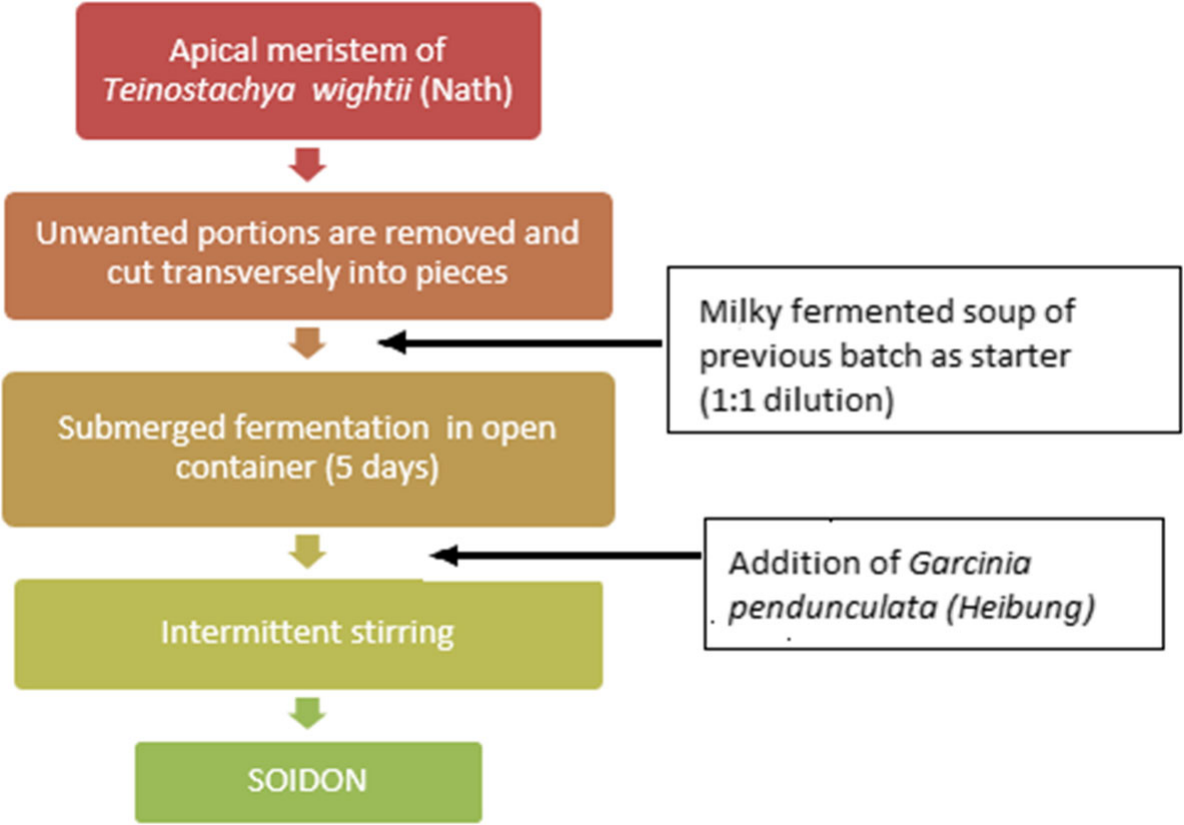
Flow chart of soidon preparation (adapted from [20])

Soidon denotes fermented bamboo shoot tips sold by Meitei women and forms a diet of inhabitants of Manipur. It is prepared in the following ways: the tip of the matured bamboo shoot (*Bambusatulda* Roxb., *Dendrocalamus giganteus* Munro, and *Melocana bambusoides* Trin, *Teinostachya wightii*) is used. In other words, the apical meristem of *Teinostachya wightii* (Nath) - bamboo shoots are taken. The lower portions and the outer casings are removed. In an earthen pot of water, the whole tips are submerged. The soijim or the sour liquid of the previous batch is added as a starter in 1:1 dilution and fermented for 3-7 days at room temperature. To increase the flavor of soidon, leaves of *Garcinia pedunculata* Roxb., known as *heibung*, locally, may be added to the fermenting vessel. Soidon can be kept for a year in a closed container, at room temperature. It is used as a curry or as a pickle [13]. The best soidon is prepared in the Bishnupur village in Manipur. This is sold in the markets by vendors [18]. The preparation of soidon is described in Fig. 3 [13, 20].

The microorganisms which are mainly found in soidon are *Lactobacillus brevis*, *Leuconostoc fallax*, and *Lactococcus lactis.* Soidon is highly nutritious in nature. It contains 92.2% moisture, pH:4.2, acidity: 0.96%, carbohydrate: 46.6%, fat: 3.1% DM, protein: 37.2%, food value: 363.1 kcal/100 gm DM, Ca: 18.5 mg/100 g, K: 245.5 mg/100 g, Na: 3.7 mg/100 g [13].

### Lung-Siej

**Fig. 4:**
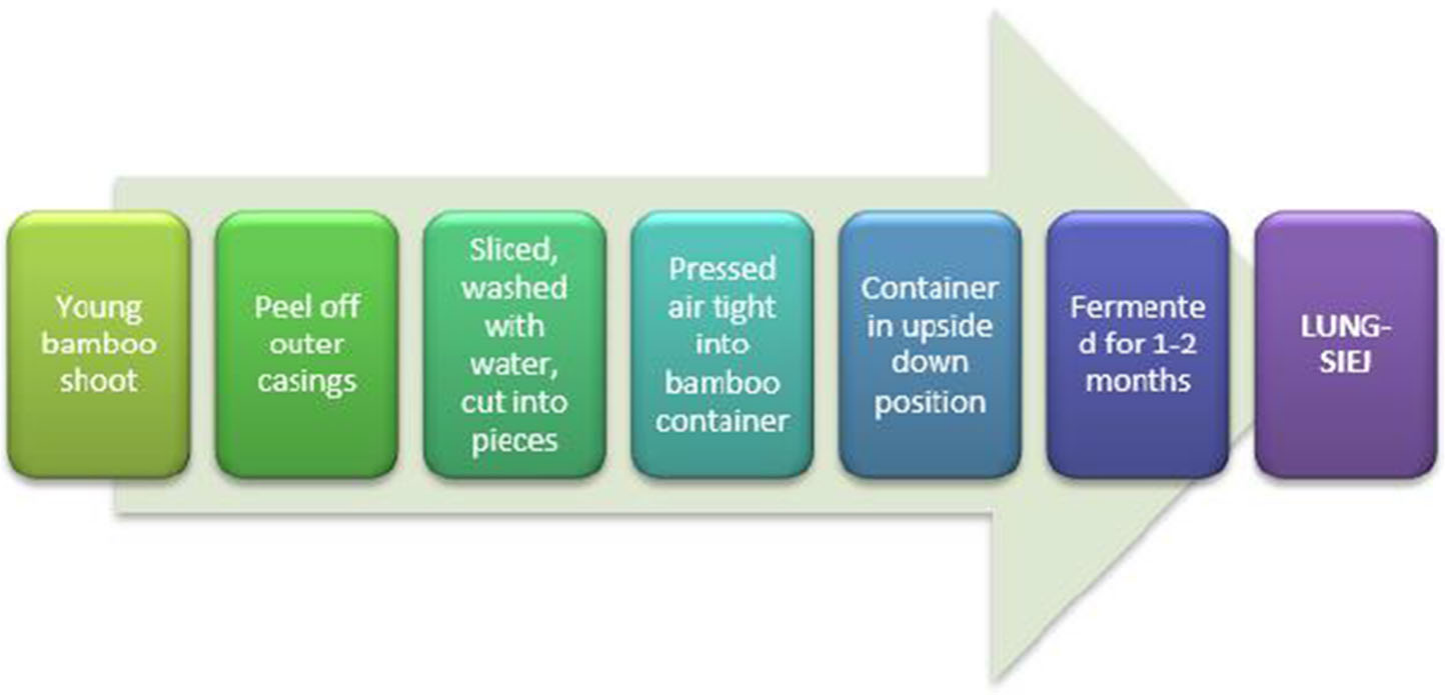
Preparation of lung-siej in Meghalaya (adapted from [11])

Lung-siej is a traditional fermented bamboo shoot food of Meghalaya made from *Dendrocalamus hamiltonii* type of bamboo found in the hills of Meghalaya. Young bamboo shoots are selected, the leaves are removed, and shoots are cut into small pieces, and put into a bamboo cylinder or in a glass bottle. Bamboo cylinders are made by cutting bamboo nodes in a way, that one side is open, and the other side is closed. Bamboo shoot slices are put into these bamboo cylinders and closed with leaves and sealed by tying the rim by grass or thread. The ends are sealed to prevent water seepage into the cylinder, which would make the shoots black and unhealthy for consumption. The bamboo cylinders are immersed near a water body upside down for 1–2 months for fermentation. Instead of bamboo cylinders, glass bottles also may be used as fermenting containers. In the case of glass bottles, sliced bamboo shoots are pressed inside it, and water is added till submerged. Then the bottle is closed with a cap and kept near the kitchen oven for 1 month. Lung-siej produced in glass bottles is better than bamboo cylinders. This is so because, lung-siej in glass bottles have a higher shelf-life, as high as 10–12 months, whereas, lung-siej prepared in bamboo cylinders have a low shelf-life of only 1–2 months. Usually, urban area people, prefer glass bottle lung-siej whereas, village people prefer bamboo cylinders. Usually, *Khasi* women are engaged in the production of lung-siej [11]. The preparation of lung-siej has been represented in Fig. 4. Lung-siej is eaten as a curry mixed with fish and meat [21]. Lactic acid bacteria (LAB) is usually found in lung-siej samples.

### Ekung

Ekung is a fermented bamboo shoot food product, ethnic to Arunachal Pradesh, produced by Nishi. It is called by different names by different dialects like *iku* by Adi and *hikku* by Apatani. Bamboo shoots locally grown (*Bambusa balcooa* Roxb. *Dendrocalamus hamiltonii* Nees. et Arn. ex Munro, *Dendrocalamus giganteus* Munro, *Bambusa tulda* Roxb., *Phyllostachys assamica* Gamble ex Brandis) are collected and the leaves are removed, and the bamboo shoots are cut into small pieces. In the forest, near a water body, a pit is dug, and the bamboo shoot pieces are washed. In the pit, the chopped bamboo pieces are laid in a bamboo basket and are covered with leaves and sealed. Heavy stones are kept for draining the water down and ferment the bamboo shoots for 1–3 months. This can be kept for a year in an air-tight container. Ekung can be cooked with meat, vegetables, or fish and is also sold in the local markets [13].

The microorganisms which are mainly present in ekung are *Lactobacillus plantarum*, *L.casei*, *L. brevis*, and *Tetragenococcus halophilus.*

The nutritional value of ekung consists of moisture: 94.7%, acidity: 0.94%, pH: 3.9, ash: 14.0% DM, food value: 363.0 kcal/100 g DM, protein: 30.1% DM, carbohydrate: 52.1% DM, fat: 3.8% DM, Ca: 35.4 mg/100 g, K: 168.6 mg/100 g, and Na: 10.9 mg/100 g [13].

**Fig. 5:**
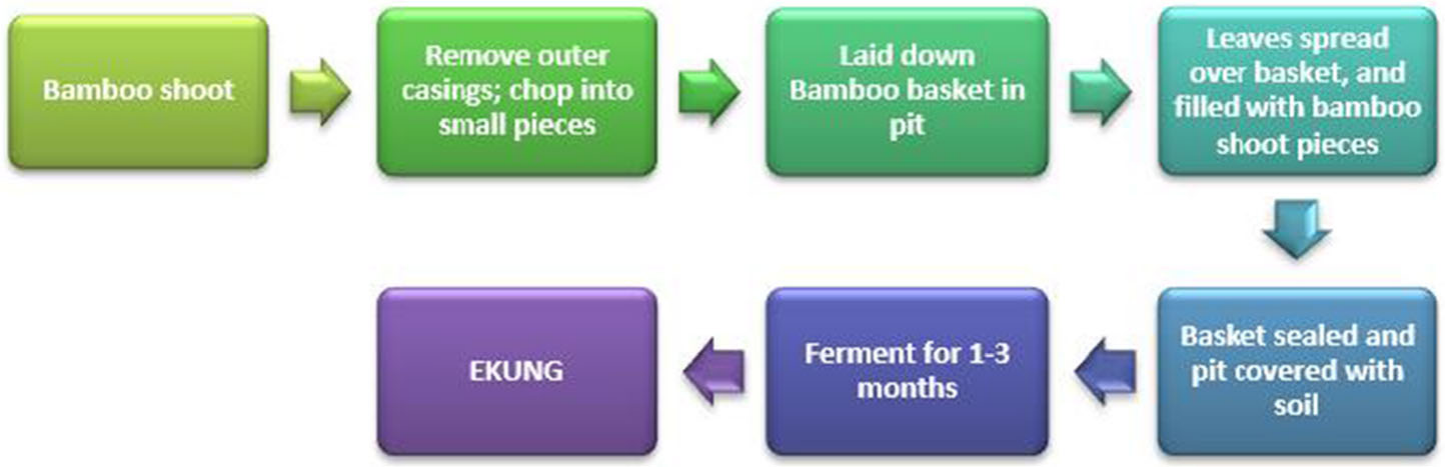
Preparation of ekung (adapted from [11])

The method of preparation of ekung has been illustrated in Fig. 5.

*Lactobacillus plantarum* in ekung, shows mechanism of action by conferring antifungal and antimicrobial properties, and also increases storage life [19].

### Eup

Eup is a dry fermented bamboo shoot of Arunachal Pradesh. The word is derived from the Nishi dialect. Eup has synonyms like Khampti call them nogom; Adi call them *ipe,* and Apatani call them *hi.* In the case of eup, bamboo shoots are cut into small pieces and fermented-like ekung, in 1–3 months. Eup is a dry food product, and bamboo shoots are chopped into pieces and dried in sun for 5–10 days till the color changes from white to chocolate brown. Eup is consumed as a curry with vegetables, fish, or meat and can be stored up to 2 years [13].

The microorganisms which are mainly found in eup are *Lactobacillus fermentum* and *L. plantarum.*

The nutritional value of eup consists of moisture: 36.8%, acidity: 0.80%, pH:4.1, ash: 18.2% DM, fat: 3.1% DM, protein: 33.6% DM, carbohydrate: 45.1% DM, food value: 342.7 kcal/100 g DM, Na: 3.4 mg/100 g, Ca: 76.9 mg/100 g, and K: 181.5 mg/100 g [13].

**Fig. 6:**
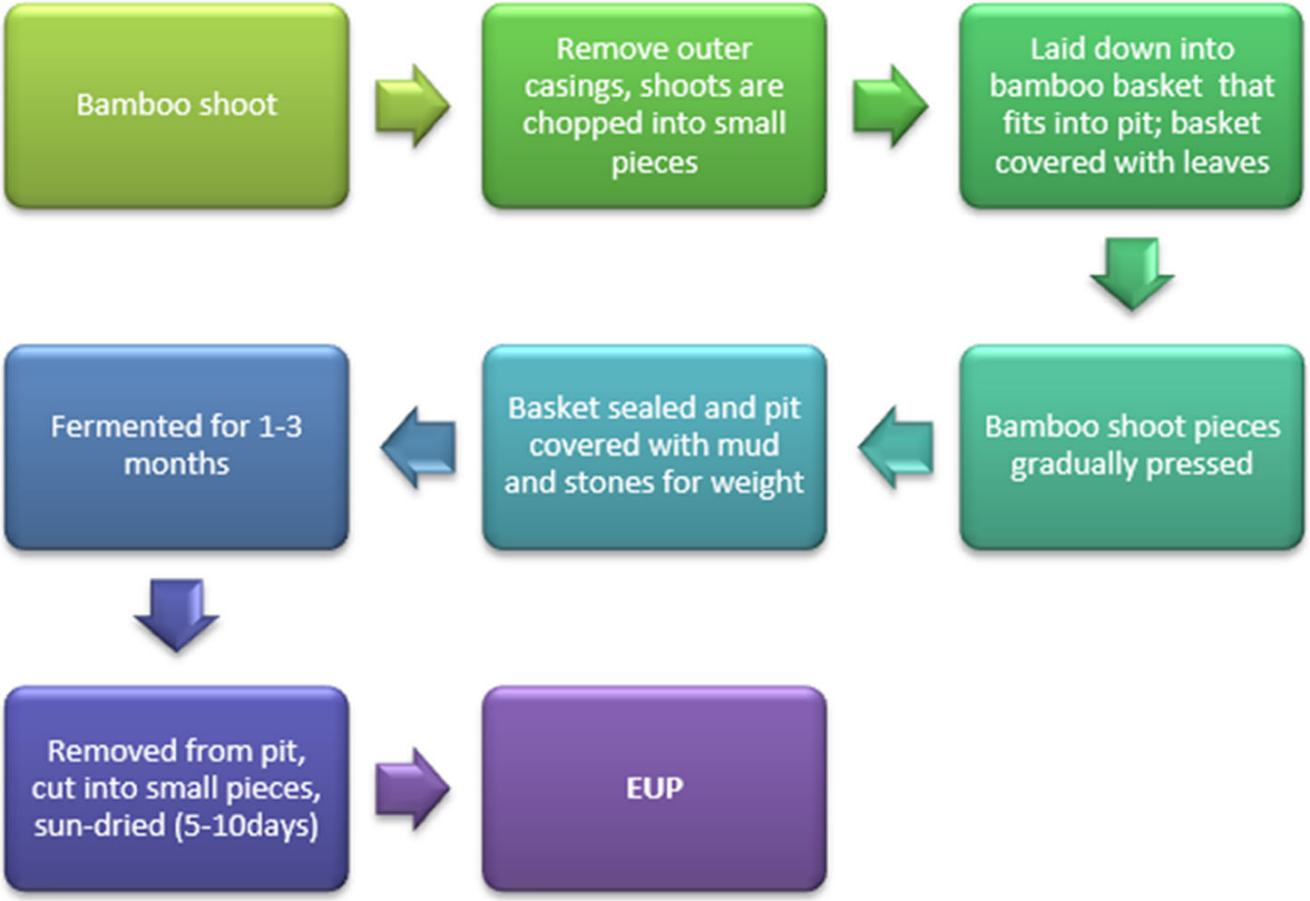
Preparation of eup in Arunachal Pradesh (adapted from [11])

The preparation of eup has been illustrated in Fig. 6.

### Hirring

Hirring is a fermented bamboo shoot product in Arunachal Pradesh made by the *Apatani* tribe. The *Nishi* call them *hitak* or *hitch.* In the production of hirring, bamboo shoots are cut longitudinally into 2–3 pieces or the shoots are flattened by crushing and are put into bamboo baskets with leaves. The baskets are placed into a pit, covered with leaves and sealed, and fermented for 1–3 months. Hirring can be consumed as a curry and is generally sold in local markets [13].

The microorganisms commonly present in hirring are *Lactobacillus plantarum* and *Lactococcus lactis* [13].

The nutritional value of hirring includes moisture: 88.8%, pH: 4.0, acidity: 0.81%, protein: 33.0% DM, carbohydrate: 49.3% DM, fat: 2.7% DM, food value: 353.5 kcal/100 g DM, Ca: 19.3 mg/100 g, K: 272.4 mg/100 g, and Na: 3.4 mg/100 g [13].

**Fig. 7:**
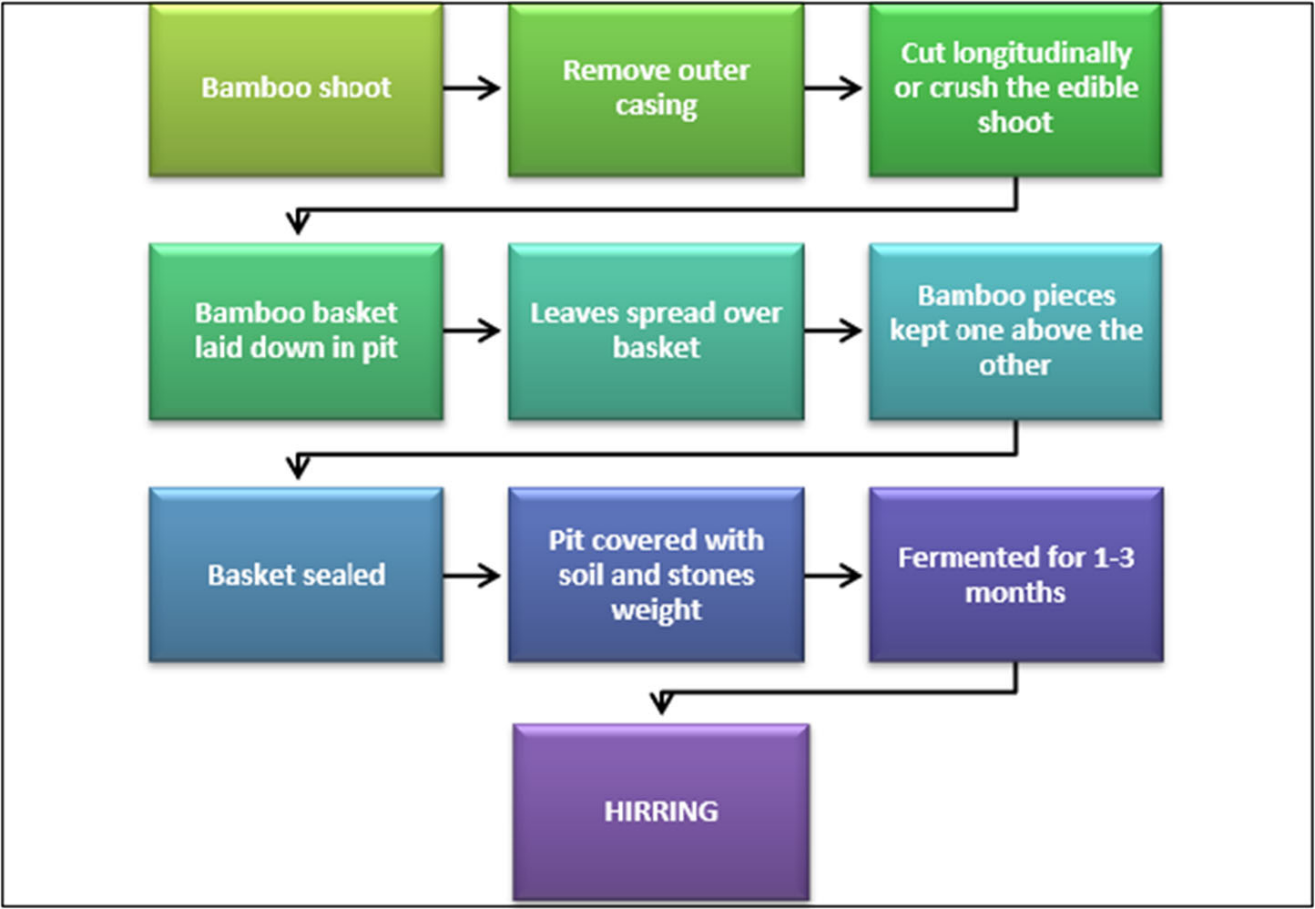
Preparation of hirring (adapted from [11])

The method of preparation of hirring is illustrated in Fig. 7.

## Advantages and Disadvantages of Fermented Bamboo Shoots

### Advantages

#### Health benefits

Bamboo shoots are fermented and consumed by tribal people of northeast India and form an integral part of their basic diet. They are rich in nutrition. They consist of a plethora of health benefits such as anti-free radicals, help in reducing cholesterol, anti-cancer, anti-oxidant, act as immune-booster, anti-aging, prevent cardiovascular diseases by protecting the heart, improve digestion, weight loss, decrease blood pressure, rich in flavones, glycosides, and it is anti-microbial, and rich in  probiotics [4]. According to Ayurveda, bamboo shoots are recommended for patients with piles and burning sensation during urination, along with honey. Bamboo shoots have low fat, high edible fiber content, and are rich in vitamins like C and E [4]. Bamboo leaves are also used for treating spasmodic disorders and for the treatment of stomach problems like killing intestinal worms, like threadworms [22]. Soibum consists of bamboo leaves and is rich in antioxidant properties [23].

#### Bamboo shoots overseas

Not only in India but also in the Philippines, Korea, and Tibet, bamboo shoots find its applications (Table 3).

**Table 3:** Applications of microorganisms commonly found in fermented bamboo shoots

Microorganisms	Applications	References
*Lactobacillus plantarum*	Can inhibit the adhesion of pathogens onto the gastrointestinal tract or urinary tract, thus inhibiting infection; also acts as probiotic; maintain intestinal microbe balance	[14]
*Lactobacillus brevis*	Produces gamma-aminobutyric acid (GABA), which has anti-depressant property	[15]
*Leuconostoc fallax*	Present in the heterofermentative stage of sauerkraut fermentation, found in the fermentation of vegetables	[16]
*Lactococcus lactis*	Food bacterium with industrial importance; used for production of industrial metabolites, enzymes, therapeutics, used as vaccine delivery system, production of heterologous plant-based and membrane-based proteins	[17]

In the Philippines, *Bambusa blumeana* (Kawayangtinik), the anti-fatigue property was reported [24]. Even in Tibet and Indo-Persia system of medicine, bamboo manna from *Bambusaarundinacea species* is considered as a beneficial tonic for respiratory disorders [25].

Apart from food and medicine, bamboo shoots are also used in the cosmetics industry. Korea has released bamboo sea salt, which is used as a cleansing agent called bamboo bath salts [26].

#### Industrial importance

Bamboo shoots have industrial importance too. They are used for the production of bioethanol. They are also used as a source of natural products like potassium, dietary fibers, carbohydrates, and vitamins. Bamboo shoots may be used to produce functional xylooligosaccharides which find application in food, biodegradable plastics or nanoparticles, and pharmaceutical industries [4].

#### Probiotics

Fermented bamboo shoots like soidon, lung-siej, ekung, soibum, and mesu are rich in microorganisms which have probiotic property. Microbes like LAB, species like *Lactobacillus plantarum* most frequently found in most fermented bamboo shoots, have potential probiotic effects along with cholesterol-lowering feature [21]. With *L. brevis*, they exhibit high hydrophobicity which indicates the ability of bacterial culture to adhere to the epithelial cell layer of the digestive tract for good colonization [6, 27].

### Disadvantages

The bamboo-based fermented foods require a large water body for its preparation and fermentation process. A long time is required for fermentation. Large volumes of bamboo shoots are chopped and cut in the forest area. This leads to deforestation.

Sometimes, the consumption of fermented bamboo shoots may lead to toxicity. The presence of cyanogenic glycosides known as taxiphyllin in bamboo shoots may lead to cyanide poisoning. This has been reported due to inhalation of hydrogen cyanide gas (HCN), which was produced from pickled bamboo shoots [8].

But, if we compare the benefits of fermented bamboo shoots with the disadvantages, it outweighs the latter and is a rich source of food value, functional food, and medicinal value.

## Future Scope

Fermented bamboo shoots have many advantages that can be used in the future. Not only do they have innumerable health benefits, but are applied in many other areas also, which are quite lucrative in the future. Consequently, the future scope of fermented bamboo shoots is very high. It can be done in the following areas:

### Industrial Applications

Fermented bamboo shoots can be used in different industrial applications. It can be used in food, pharmaceuticals, biofuels, to name a few. In the food industry, it has high growth potential. It can be used as functional and healthy foods, as medicines, and as a source of bioactive compounds. It is a niche for various lactic acid bacteria which act as probiotics. Though the preparation of fermented bamboo shoots is local and prepared by tribal people of Northeast India, they have great scope and value among the food source of plant origin in the Asian countries. It may be just “supporting sticks” for poor people, and also a tasty cuisine. In the international market, fermented bamboo shoots form an important aspect in terms of nutrition, health, and medicinal property due to probiotic microflora [4].

In the biofuel industry, it can be used to produce ethanol or methane. It has been used to produce bioethanol, which is a source of carbohydrates, potassium, vitamins, and dietary fibers. Bio-methane can be produced from bio-ethanol which has high holocellulose content and biomass yield [4].

### Economic Value

Fermented bamboo shoots have high economic importance [4]. Usually, the fermented bamboo shoots are produced by the local and tribal people of Northeast India. It is limited to the local market and sold by local women like the *Meiteis.* However, due to numerous food and health benefits, the medicinal values, it can be sold out in the international market also, to other countries [24–26]. Hence, the fermented bamboo shoots are profitable and have high economic importance in the future. 

In our opinion, fermented bamboo shoots are very healthy and have high future scope in the fields of food, pharmaceutical, and other industries, due to their plethora of health benefits, medicinal properties, and probiotic nature. It should be publicized in the national and international markets, and not be hidden only in the local tribal markets. More care should be taken in the area of preservation of fermented bamboo shoots for a longer time so that the shelf- life can be increased.

## Conclusion

Fermented bamboo shoots are a storehouse of microorganisms, many of them being probiotics in nature. When consumed, they produce a lot of health benefits, such as anti-oxidant, anti-cancer, reduces blood pressure, prevents cardiovascular diseases, weight loss, to name a few. Besides these, they can also be applied in the industries, especially in the food, pharmaceutical, and biofuel industries. They have economic value and the benefits are far more than the disadvantages, that is why, they are called the “green gold,” of India.
